# Left Ventricle Mass Index, a Confounding Variable of Global Longitudinal Strain to be Noticed

**DOI:** 10.36660/abc.20190708

**Published:** 2020-01

**Authors:** Eduardo Thadeu de Oliveira Correia, Letícia Mara dos Santos Barbetta

**Affiliations:** 1 Hospital Universitário Antônio Pedro, Niterói, RJ - Brazil

**Keywords:** Ventricular Function, Left, Cardiomyopathy, Hypertrophic, Hypertension, Strain, Heart Failure, Hypertrophy, Left Ventricular

**Dear Editor,**

We read with great interest the article entitled as “Strain Analysis of Left Ventricular Function in the Association of Hypertrophic Cardiomyopathy and Systemic Arterial Hypertension”. In this paper, the authors evaluated the global longitudinal strain (GLS) of the left ventricle (LV) in two distinct groups: patients with hypertrophic cardiomyopathy (HCM) and patients with HCM and systemic arterial hypertension (SAH) and demonstrated that GLS was lower in the second group. This important finding may indicate greater impairment of LV function in patients with SAH and HCM.^1^

However, despite these findings, it is important to notice the impact of the LV mass on the GLS, unfortunately not reported by the authors. A previous study by Soufi Taleb Bendiab et al.^2^ showed that hypertensive patients with LV hypertrophy had a reduced GLS.^2^ Moreover, in the study of Lópes-Candales et al.^3^ patients with increased LV mass had a significant reduced GLS.^3^ From a mechanical point of view of a physiological heart model validated by echocardiography, the volume of heart tissue is constant throughout the cardiac cycle, since it is incompressible.^4^ In that way, patients with increased LV thickness would have less longitudinal myocardial deformation.^3^

Since the population of this study were patients with hypertrophic cardiomyopathy with or without hypertension, the measurement of LV mass becomes even more important to report an accurate multivariate analysis and to measure the impact of this important confounder on the study’s conclusions.

## References

[1] Gil TGP, Castier MB, Gondar AFP, Sales AF, Santos M de O, Lima FC da S de, Mourilhe-Rocha R (2019). Strain Analysis of Left Ventricular Function in the Association of Hypertrophic Cardiomyopathy and Systemic Arterial Hypertension. Arq Bras Cardiol.

[2] Soufi Taleb Bendiab N, Meziane-Tani A, Ouabdesselam S, Methia N, Latreche S, Henaoui L (2017). Factors associated with global longitudinal strain decline in hypertensive patients with normal left ventricular ejection fraction. Eur J Prev Cardiol.

[3] López-Candales A (2014). Automated functional imaging for assessment of left ventricular mechanics in the presence of left ventricular hypertrophy. Echocardiography.

[4] Ghosh E, Shmuylovich L, Kovács SJ (2010). Vortex formation time-to-left ventricular early rapid filling relation: model-based prediction with echocardiographic validation. J Appl Physiol.

